# T2/FLAIR-mismatch sign for noninvasive detection of IDH-mutant 1p/19q non-codeleted gliomas: validity and pathophysiology

**DOI:** 10.1093/noajnl/vdaa004

**Published:** 2020-01-10

**Authors:** Martha Foltyn, Karen Natalia Nieto Taborda, Ulf Neuberger, Gianluca Brugnara, Annekathrin Reinhardt, Damian Stichel, Sabine Heiland, Christel Herold-Mende, Andreas Unterberg, Jürgen Debus, Andreas von Deimling, Wolfgang Wick, Martin Bendszus, Philipp Kickingereder

**Affiliations:** 1 Department of Neuroradiology, University of Heidelberg Medical Center, Heidelberg, Germany; 2 Department of Neuropathology, University of Heidelberg Medical Center, Heidelberg, Germany; 3 Department of Neurosurgery, University of Heidelberg Medical Center, Heidelberg, Germany; 4 Department of Radiation Oncology, University of Heidelberg Medical Center, Heidelberg Institute of Radiation Oncology and National Center for Radiation Research in Oncology, Heidelberg, Germany; 5 Division of Molecular and Translational Radiation Oncology, National Center for Tumor Diseases, Heidelberg University Hospital and DKFZ, Heidelberg, Germany; 6 Clinical Cooperation Unit Neuropathology, German Cancer Consortium (DKTK), German Cancer Research Center (DKFZ), Heidelberg, Germany; 7 Neurology Clinic, University of Heidelberg Medical Center, Heidelberg, Germany; 8 Clinical Cooperation Unit Neuro-oncology, DKTK, DKFZ, Heidelberg, Germany

**Keywords:** biomarkers, glioma, isocitrate dehydrogenase, magnetic resonance imaging

## Abstract

**Background:**

This study aimed to assess the validity and pathophysiology of the T2/FLAIR-mismatch sign for noninvasive identification of isocitrate dehydrogenase (IDH)-mutant 1p/19q non-codeleted glioma.

**Methods:**

Magnetic resonance imaging scans from 408 consecutive patients with newly diagnosed glioma (113 lower-grade gliomas and 295 glioblastomas) were evaluated for the presence of T2/FLAIR-mismatch sign by 2 independent reviewers. Sensitivity, specificity, accuracy, positive predictive value (PPV), and negative predictive value (NPV) were calculated to assess the performance of the T2/FLAIR-mismatch sign for identifying IDH-mutant 1p/19q non-codeleted tumors. An exploratory analysis of differences in contrast-enhancing tumor volumes, apparent diffusion coefficient (ADC) values, and relative cerebral blood volume (rCBV) values in IDH-mutant gliomas with versus without the presence of a T2/FLAIR-mismatch sign (as well as analysis of spatial differences within tumors with the presence of a T2/FLAIR-mismatch sign) was performed.

**Results:**

The T2/FLAIR-mismatch sign was present in 12 cases with lower-grade glioma (10.6%), all of them being IDH-mutant 1p/19q non-codeleted tumors (sensitivity = 10.9%, specificity = 100%, PPV = 100%, NPV = 3.0%, accuracy = 13.3%). There was a substantial interrater agreement to identify the T2/FLAIR-mismatch sign (Cohen’s kappa = 0.75 [95% CI, 0.57–0.93]). The T2/FLAIR-mismatch sign was not identified in any other molecular subgroup, including IDH-mutant glioblastoma cases (*n* = 5). IDH-mutant gliomas with a T2/FLAIR-mismatch sign showed significantly higher ADC (*P* < .0001) and lower rCBV values (*P* = .0123) as compared to IDH-mutant gliomas without a T2/FLAIR-mismatch sign. Moreover, in IDH-mutant gliomas with T2/FLAIR-mismatch sign the ADC values were significantly lower in the FLAIR-hyperintense rim as compared to the FLAIR-hypointense core of the tumor (*P* = .0005).

**Conclusions:**

This study confirms the high specificity of the T2/FLAIR-mismatch sign for noninvasive identification of IDH-mutant 1p/19q non-codeleted gliomas; however, sensitivity is low and applicability is limited to lower-grade gliomas. Whether the higher ADC and lower rCBV values in IDH-mutant gliomas with a T2/FLAIR-mismatch sign (as compared to those without) translate into a measurable prognostic effect requires investigation in future studies. Moreover, spatial differences in ADC values between the core and rim of tumors with a T2/FLAIR-mismatch sign potentially reflect specific distinctions in tumor cellularity and microenvironment.

**Key Points**

- The T2/FLAIR-mismatch sign has high specificity but low sensitivity in lower-grade gliomas.
- The T2/FLAIR-mismatch sign is most likely not useful in glioblastoma.
- The T2/FLAIR-mismatch sign is associated with variations in tumor cellularity and angiogenesis.

**Importance of the Study**

This study confirms the high specificity of the previously described T2/FLAIR-mismatch sign for noninvasive identification of IDH-mutant 1p/19q non-codeleted gliomas. By performing analysis of advanced magnetic resonance imaging parameters, significantly higher ADC and lower rCBV values were found for IDH-mutant gliomas with a T2/FLAIR-mismatch sign (as compared to those without the sign). Whether this phenomenon translates into a clinically measurable prognostic effect requires further investigation in future studies. Moreover, spatial differences in ADC values were identified between the FLAIR-hypointense core and the FLAIR-hyperintense rim of tumors with a T2/FLAIR-mismatch sign. This sheds light on the pathophysiological correlates of the T2/FLAIR-mismatch sign with lower ADC values in the FLAIR-hyperintense rim potentially reflecting spatial differences in the cellularity and microenvironment of these tumors. Overall, given its high specificity, the T2/FLAIR-mismatch sign can aid in the initial workup of patients with suspected lower-grade gliomas to better guide treatment decisions and patient counsel.

Diffuse gliomas are the most common adult primary brain tumors. The classification and grading of diffuse gliomas has evolved over time and gliomas are now—after the introduction of the most recent 2016 World Health Organization (WHO) Classification of Tumors of the Central Nervous System—classified based not only on histopathologic appearance but also on well-established hallmark molecular parameters.^1^ Specifically, hallmark molecular features such as isocitrate dehydrogenase (IDH) mutation status, expression of transcription regulator *ATRX*, 1p/19q co-deletion status, or K27M mutations in the histone H3 gene *H3F3A* were included in the classification, to identify more biologically homogeneous and narrowly defined diagnostic entities for greater diagnostic accuracy and improved patient management. It has previously been shown that noninvasive signatures on magnetic resonance imaging (MRI) can serve as surrogates for some of these molecular alterations and thus may steer the preoperative differential diagnosis of these tumors and potentially aid treatment decision processes.^2^ Recent efforts primarily focused on identifying those imaging surrogates through radiomics-based imaging analysis.^3^ However, these sophisticated analyses are poorly standardized and reproducible, requiring further validation.^4^ Moreover, they are also not readily available in the clinical routine. Complementary efforts that aim to identify easily discernible imaging surrogates for molecular features on standard MRI may therefore be of great value in the management of patients with glioma.^5–8^ A prime example in this respect is the recent identification of the T2/FLAIR-mismatch sign on standard MRI which was found to be a highly specific imaging biomarker (with reported specificity up to 100%) for identifying adult patients with IDH-mutant 1p/19q non-codeleted lower-grade gliomas.^5,6,9–11^ This noninvasive imaging marker is easily discernible on standard routine MRI sequences and is defined by a completely or almost completely homogeneous hyperintense signal on T2-w sequence and simultaneously completely or almost completely homogeneous hypointense signal on FLAIR sequence with a complete or near-complete hyperintense peripheral rim on FLAIR.^5^ So far, there is no data available regarding the biological underpinning of the T2/FLAIR-mismatch sign as well as regarding the value of the T2/FLAIR-mismatch sign in patients with glioblastoma.

The aim of this study was to assess the presence of the T2/FLAIR-mismatch sign for noninvasive identification of IDH-mutant (and 1p/19q non-codeleted) tumors in an unselected cohort of patients with glioma. Specifically, we aimed to validate the previously described T2/FLAIR-mismatch sign in patients with lower-grade glioma^5,6^ and additionally investigate its potential value in patients with glioblastoma. Moreover, we investigated the pathophysiology of the T2/FLAIR-mismatch sign by performing an exploratory analysis of contrast-enhancing tumor volumes, apparent diffusion coefficient (ADC) values from diffusion-weighted MRI (DWI), and relative cerebral blood volume (rCBV) values from dynamic susceptibility-weighted contrast MRI (DSC-MRI) between IDH-mutant gliomas with versus without the presence of the T2/FLAIR-mismatch sign. Moreover, in IDH-mutant gliomas with the presence of the T2/FLAIR mismatch, we performed a spatial analysis of ADC and rCBV values comparing values between the FLAIR-hypointense core and the FLAIR-hyperintense rim.

## Materials and Methods

Retrospective evaluation of imaging data was approved by the local ethics committee of the University of Heidelberg, and the requirement to obtain informed consent was waived (S-320/2012 and S-784/2018). All patients with newly diagnosed and histologically confirmed lower-grade glioma (ie, diffuse astrocytoma WHO °II, anaplastic astrocytoma WHO °III, diffuse oligodendroglioma WHO °II, and anaplastic oligodendroglioma WHO °III)^12^ or glioblastoma (WHO °IV) at the Heidelberg University Hospital (Heidelberg, Germany) between February 2009 and March 2018 were screened. All diagnosis were based on the 2016 WHO Classification of Tumors of the Central Nervous System.^1^ Inclusion criteria were (1) availability of key molecular parameters (IDH mutation status and 1p/19q co-deletion status) obtained from tissue specimens of the initial surgery (all patients underwent surgical resection or biopsy at the Department of Neurosurgery at Heidelberg University Hospital and tissue specimens were gathered according to the research procedures approved by the Institutional Review Board at the Medical Faculty Heidelberg); furthermore, (2) availability of standardized pretreatment MRI performed at the Department of Neuroradiology at Heidelberg University Hospital.

A total of 444 patients (*n* = 309 with glioblastoma and *n* = 135 with lower-grade glioma) were screened for inclusion. Fourteen patients with glioblastoma were excluded because of multiple lesions (*n* = 13) or incomplete MRI acquisition (no T2-w sequence) (*n* = 1). Twenty-two patients with lower-grade glioma were excluded because of incomplete MRI protocol (no T2-w or FLAIR sequences) (*n* = 15), partial 1p/19q deletion (*n* = 3), multiple lesions (*n* = 3) or because the lesion was not well visualized (*n* = 1). Ultimately, a total of 408 were included in the present study: 295 patients with glioblastoma and 113 with lower-grade glioma (Supplementary Table 1).

### Magnetic Resonance Imaging

MR images were acquired in the routine clinical workup using a 3 T MR system (Magnetom TIM Trio/Prisma Fit, Verio or Skyra, Siemens Healthineers) with a 12-channel head-matrix coil. Briefly, the protocol included T1-weighted 3D MP-RAGE images both before (T1) and after (cT1) administration of a 0.1 mmol/kg dose of gadoterate meglumine (DOTAREM) as well as axial FLAIR and axial T2-w images, as described previously.^13^ Sequence parameters for T1 and cT1 MP-RAGE (3D sagittal or axial) were as follows: TI = 900–1100 ms, TE = 3–4 ms, TR = 1710–2250 ms and FA = 15°; for T2 (2D, axial): TE = 85–88 ms; TR = 2740–5950 ms; section thickness, 5 mm; spacing, 5.5mm; for FLAIR (2D, axial): TI = 2400–2500 ms; TE = 85–135 ms; TR = 8500–10 000 ms; section thickness, 5 mm; spacing, 5.5 mm.

For the subset of patients with the presence of a T2/FLAIR-mismatch sign analysis of DWI and DSC-MRI was performed. DWI was performed using a single-shot spin-echo (SE) echo-planar sequence with the following parameters: echo time (TE) = 90 ms, repetition time (TR) = 5300 ms, flip angle (FA) = 90°, slice thickness = 5 mm. Diffusion sensitizing gradients were applied sequentially in the x, y, and z directions with *b*-values of 0 and 1200 s/mm^2^ and corresponding ADC maps were generated by the Syngo software of the MRI scanner (Siemens Healthineers). Before dynamic imaging, a 0.1 mmol/kg pre-bolus dose of gadoterate meglumine was administered to diminish T1 effects that might result from agent extravasation. DSC-MRI was obtained with a T2*-weighted gradient echo-planar sequence during the administration of a bolus of a standard dose (0.1 mmol/kg) of intravenous gadoterate meglumine. Twenty-six to 28 5-mm-thick sections were acquired with the following parameters: 2220/36, fat suppression, 90° flip angle, 240 × 240 mm^2^ field of view, 128 × 128 matrix. In total, 50–75 dynamic measurements were obtained. Postprocessing of DSC-MRI was performed with dedicated software (Olea Sphere v 2.3; Olea Medical). Individual temporal volumes of DSC-MRI were corrected for motion artifacts with a rigid-body coregistration method. The arterial input function was automatically determined by using cluster analysis techniques, and deconvolution of the arterial input function was performed with a time-insensitive block-circulant singular value decomposition.^14,15^ Mathematic leakage-corrected, whole-brain rCBV maps were computed by voxel-wise division of the area under the concentration–time curve of the respective voxel by the area under the arterial input function (assuming 100% partial blood volume of arterial input function voxels).^16^

### Image Analysis

MRI examinations were independently analyzed by 2 investigators (M.F., a radiology resident with 3 years of experience and K.N.N.T., a radiology resident with 5 years of experience). Both were blinded to clinical history, molecular classification, and histopathologic diagnosis. The presence of a T2/FLAIR-mismatch sign was identified if the following criteria were fulfilled: (1) presence of a homogenous or near-homogenous hyperintense signal intensity on T2-w images, (2) presence of homogenous or near-homogenous hypointense signal intensity on FLAIR, and (3) presence of a complete or a near-complete hyperintense rim on FLAIR.^5^ Discrepancies in the ratings for the presence of a T2/FLAIR-mismatch sign between the 2 investigators were resolved through a consensus discussion with a third investigator (P.K., a board-certified radiologist with 7 years of experience).

For patients with IDH-mutant gliomas comparison of contrast-enhancing tumor volumes (available for 115/155 [100%] IDH-mutant gliomas) as well as ADC values (available for a subset of 99/115 [86%] IDH-mutant gliomas) and rCBV values (available for a subset of 75/115 [65%] IDH-mutant gliomas) was performed between those cases with versus without the presence of a T2/FLAIR-mismatch sign. In addition, for patients with IDH-mutant gliomas who present the T2/FLAIR-mismatch sign, an additional exploratory analysis of spatial differences in ADC values (available for 12/12 [100%] cases with the presence of a T2/FLAIR-mismatch sign) and rCBV values (available for 9/12 [75%] cases with the presence of a T2/FLAIR-mismatch sign) between the FLAIR-hypointense core and the FLAIR-hyperintense rim of the tumor was performed. For all these cases postprocessing of MRI data included brain extraction, image registration, T1-subtraction mapping, and tumor segmentation as described previously.^13,17^ Specifically, separate segmentation masks for (1) the whole tumor, (2) the contrast-enhancing tumor volume as well as (3) the FLAIR hyperintense rim and (4) the hyperintense tumor on T2-w images were generated using ITK-SNAP (www.itksnap.org^18^).^19,20^

### Molecular Analysis

IDH mutation status and 1p/19q co-deletion status were obtained in all 295 glioblastoma cases and 67/113 lower-grade glioma cases (59%) from the Illumina Infinium Human Methylation 450K or EPIC array (Illumina) as described previously.^21^ For the remaining 46/113 lower-grade glioma cases (41%) IDH mutation status was assessed with immunohistochemistry (IHC) for IDH1-R132H and DNA sequencing for IHC-negative cases, whereas detection of chromosome arms 1p and 19q deletions was assessed with fluorescence in situ hybridization.^22^

### Statistical Analysis

Statistical analysis was performed using R (R version 3.5.1, R Foundation for Statistical Computing). The interrater agreement was calculated using the Cohen’s kappa coefficient (*k*), with a value ranging from 0.00 to 1.00 and interpreted for *k* values 0.81–1.00 as an almost perfect agreement, 0.61–0.80 as substantial agreement, 0.41–0.60 as moderate agreement, 0.21–0.40 as fair agreement, and ≤0.20 as slight agreement.^23^ The performance of the T2/FLAIR-mismatch sign for identifying IDH-mutant 1p/19q non-codeleted tumors was evaluated by calculating its sensitivity, specificity, and accuracy, as well as positive and negative predictive values (PPV and NPV). A Wilcoxon signed-rank test was performed to evaluate the difference in ADC values, rCBV values, and contrast-enhancing tumor volumes between IDH-mutant gliomas with versus without a T2/FLAIR-mismatch sign. Moreover, a Wilcoxon matched-pairs signed-rank test was performed to evaluate the spatial differences in ADC and rCBV values between the FLAIR-hypointense core versus the FLAIR-hyperintense rim of IDH-mutant gliomas with a T2/FLAIR-mismatch sign. *P*-values <.05 were considered significant.

## Results

**Figure 1.**
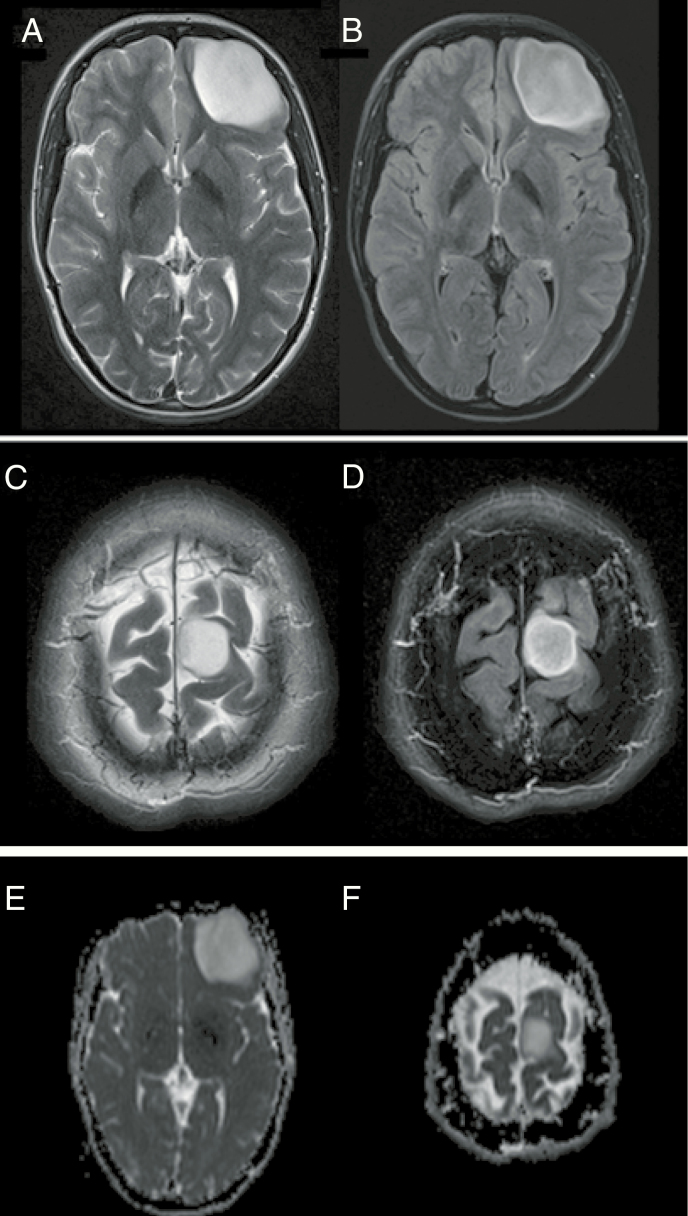
Two cases with the presence of a T2/FLAIR-mismatch sign and their corresponding ADC image (E and F). (A and B) IDH-mutant 1p/19q non-codeleted anaplastic astrocytoma (WHO °III) in a 31-year-old man and (C and D) IDH-mutant 1p/19q non-codeleted anaplastic astrocytoma (WHO °III) in a 56-year-old woman. Both cases show tumors located in the left frontal lobe with a homogeneously hyperintense signal on T2-w images (left side), coupled with a homogeneously hypointense signal on FLAIR images except for a small hyperintense peripheral rim (right side).

Supplementary Table 1 displays the characteristics of the patients included in the present study. Briefly, the study cohort consisted of 28 cases (6.9%) with diffuse astrocytoma IDH-mutant (WHO °II), 38 cases (9.3%) with anaplastic astrocytoma IDH-mutant (WHO °III), 3 cases (0.7%) with anaplastic astrocytoma IDH-wildtype (WHO °III), 33 cases (8.1%) with oligodendroglioma IDH-mutant and 1p/19q codeleted, 11 cases (2.7%) with anaplastic oligodendroglioma IDH-mutant and 1p/19q codeleted, 5 cases (1.2%) with glioblastoma IDH-mutant, 282 cases (69.1%) with glioblastoma IDH-wildtype, and 8 cases (2%) with diffuse midline glioma H3-K27M-mutant. The T2/FLAIR-mismatch sign was detected in 12/113 cases with lower-grade glioma (10.6%), all of them were IDH-mutant (Figure 1), 1p/19q non-codeleted tumors (6 cases with diffuse astrocytoma, IDH mutant [WHO °II] and 6 cases with anaplastic astrocytoma, IDH-mutant) and in 0/295 cases with glioblastoma (0.0%) (Table 1).

**Table 1.** Study Population Distributed by Integrated Diagnosis and Presence or Absence of T2/FLAIR-Mismatch Sign

Tumor Entity	T2/FLAIR-Mismatch Sign	
	Positive	Negative
Diffuse astrocytoma IDH-mut. (WHO °II)	6	22
Anaplastic astrocytoma IDH-mut. (WHO °III)	6	32
Anaplastic astrocytoma IDH-wildtype (WHO °III)	0	3
Oligodendroglioma IDH-mut. 1p/19q codeleted (WHO °II)	0	33
Anaplastic oligodendroglioma IDH- mut. 1p/19q codeleted (WHO °III)	0	11
Glioblastoma IDH-wildtype (WHO °IV)	0	282
Glioblastoma IDH-mut. (WHO °IV)	0	5
Diffuse midline glioma H3-K27M-mut. (WHO °IV)	0	8
Total	12	396

**Figure 2.**
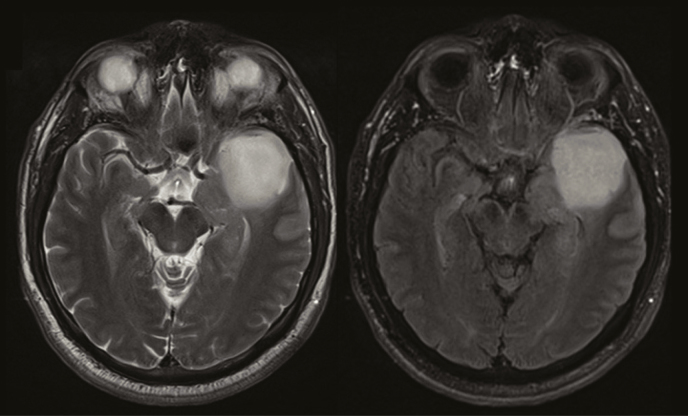
Absence of a T2/FLAIR-mismatch sign in a 36-year-old woman with a left temporal IDH-mutant 1p/19q non-codeleted anaplastic astrocytoma (WHO °III). The tumor demonstrates a near-complete homogeneous hyperintense signal on both T2-w and FLAIR images (right). No peripheral hyperintense rim is visible on FLAIR images.

There was a substantial interrater agreement for the assessment of the T2/FLAIR-mismatch sign, with a Cohen’s *k* coefficient of 0.75 (95% CI, 0.57–0.93). Specifically, concordant ratings were achieved for 401/408 cases (98.3%) whereas the discordant ratings for the remaining 7/408 cases (1.7%) were resolved by consensus. Among patients with lower-grade glioma, the T2/FLAIR-mismatch sign had a high specificity of 100% and 100% of PPV for detecting IDH-mutant (1p/19q non-codeleted) tumors, with a sensitivity of 10.9%, NPV of 3.0%, and accuracy of 13.3%. The T2/FLAIR-mismatch sign was not identified in any other molecular subgroup, especially not in any of the IDH-mutant (1p/19q non-codeleted) glioblastoma cases (Figure 2).

**Figure 3.**
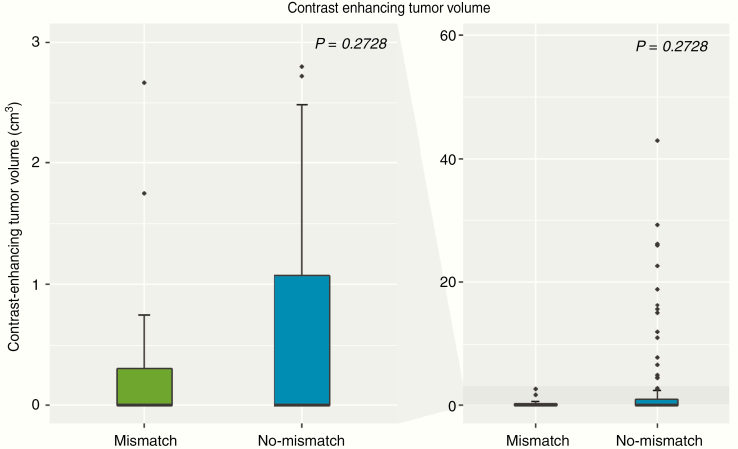
Boxplot of contrast-enhancing tumor volumes (cm^3^) comparing patients with IDH-mutant gliomas who present the T2/FLAIR-mismatch sign (green color) and who did not (blue color). The left side of the figure zooms to the range of 0–3 cm^3^, whereas the right side shows the full range of data (0–60 cm^3^). There was no significant difference in contrast-enhancing tumor volumes between patients who present the T2/FLAIR-mismatch sign and patients who did not (*P* = .2728).

**Figure 4.**
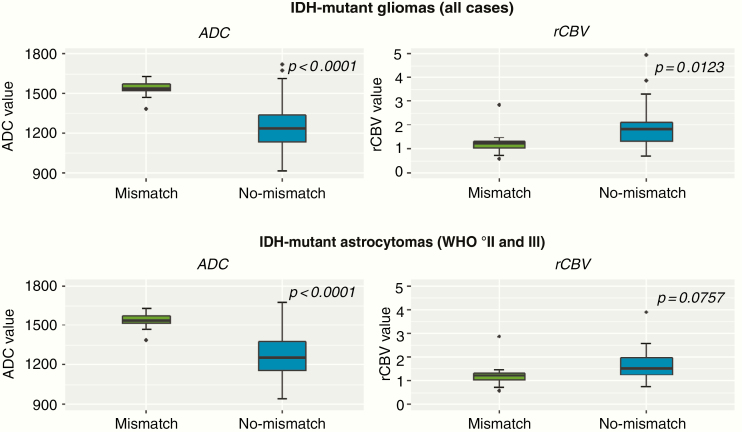
Boxplot of mean ADC and rCBV values comparing patients with IDH-mutant gliomas who present the T2/FLAIR-mismatch sign (green color) and those who did not (blue color). Analysis of all IDH-mutant gliomas (ie, including astrocytoma, oligodendroglioma, and glioblastoma cases—top row) and separate analysis for the subset of patients with IDH-mutant astrocytomas (bottom row) were performed. The median ADC values were significantly higher in both IDH-mutant gliomas (*P* < .0001) and in the subset of IDH-mutant astrocytomas (*P* < .0001) without the T2/FLAIR-mismatch sign as compared those with a T2/FLAIR-mismatch sign (left column). The median rBCV values were significantly lower in IDH-mutant gliomas (*P* = .0123) with the presence of a T2/FLAIR-mismatch sign, whereas only borderline significance was found within the subset of IDH-mutant astrocytomas (*P* = .0757) (right column).

There was no significant difference in contrast-enhancing tumor volumes between IDH-mutant gliomas with versus without a T2/FLAIR-mismatch sign (*P* = .2728; Figure 3). Differences in both ADC and rCBV values were found for IDH-mutant gliomas with the presence of a T2/FLAIR-mismatch sign as compared to IDH-mutant gliomas without the presence of a T2/FLAIR-mismatch sign (*P* < .0001 and *P* = .0123, respectively) (Figure 4, top row). Specifically, median ADC values were significantly higher for those patients with T2/FLAIR-mismatch sign (median ADC of 1536 × 10^–6^ mm^2^/s (interquartile range [IQR], 1518–1572 × 10^–6^ mm^2^/s)) as compared to those without a T2/FLAIR-mismatch sign (median ADC of 1234 × 10^–6^ mm^2^/s [IQR, 1132–1334 × 10^–6^ mm^2^/s]). In addition, median rCBV values were significantly lower for those patients with T2/FLAIR-mismatch sign (median rCBV of 1.22 [IQR, 1.03–1.32]) as compared to those without a T2/FLAIR-mismatch sign (median rCBV of 1.81 [IQR, 1.30–2.13]). To exclude a potential confounding effect of IDH-mutant oligodendrogliomas and glioblastomas, all of which demonstrated no T2/FLAIR-mismatch sign in our study and which are known to frequently demonstrate lower ADC and higher rCBV values as compared to IDH-mutant astrocytomas,^24–26^ additional analysis was performed only for the subset of patients with IDH-mutant astrocytomas. Thereby, similar results were obtained with significantly higher median ADC values for IDH-mutant astrocytomas with the presence of a T2/FLAIR-mismatch sign (median ADC of 1536 × 10^–6^ mm^2^/s [IQR, 1518–1572 × 10^–6^ mm^2^/s]) as compared to those without a T2/FLAIR-mismatch sign (median ADC of 1256 × 10^–6^ mm^2^/s [IQR, 1157–1376 × 10^–6^ mm^2^/s]) (*P* < .0001; Figure 4, bottom row). The median rCBV values for IDH-mutant astrocytoma were also lower for those with the presence of a T2/FLAIR-mismatch sign (median rCBV of 1.22 [IQR, 1.03–1.32]) as compared to those without a T2/FLAIR-mismatch sign (median rCBV of 1.52 [IQR, 1.24–1.97]), although the difference did not reach statistical significance (*P* = .0757; Figure 4, bottom row).

**Figure 5.**
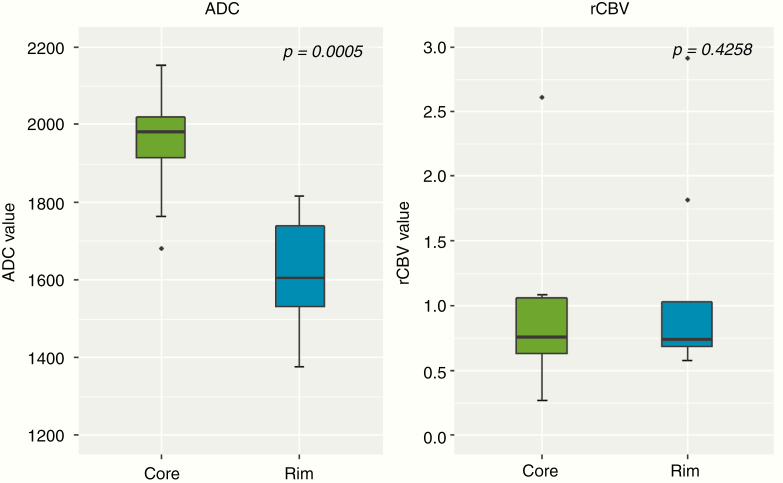
Boxplot of mean ADC and rCBV values comparing the FLAIR-hypointense core (green color) versus the FLAIR-hyperintense rim (blue color) in patients with a T2/FLAIR-mismatch sign. ADC values were significantly lower in the rim as compared to the core (*P* = .0005) whereas there was no significant difference in rCBV values (*P* = .4258).

Exploratory analysis of spatial differences in ADC and rCBV values between the FLAIR-hyperintense rim and the FLAIR-hypointense core of patients with a T2/FLAIR-mismatch sign showed that median ADC values were significantly lower in the FLAIR-hyperintense rim (median ADC of 1604 × 10^–6^ mm^2^/s [IQR, 1532–1738 × 10^–6^ mm^2^/s]) as compared to the FLAIR-hypointense core (median ADC of 1982 × 10^–6^ mm^2^/s [IQR, 1916–2021 × 10^–6^ mm^2^/s]) (*P* = .0005; Figure 5, left side). In contrast, there was no significant difference in rCBV values between the FLAIR-hyperintense rim versus FLAIR-hypointense core of patients with a T2/FLAIR-mismatch sign (*P* = .4258; Figure 5, right side).

## Discussion

Our study confirms that the T2/FLAIR-mismatch sign is a highly specific but relatively insensitive imaging biomarker for identifying IDH-mutant (1p/19q non-codeleted) lower-grade gliomas from standard anatomical MRI with a high interrater agreement.^5,6,10,11^ In contrast to previous studies, however, we did not only focus on lower-grade gliomas but also investigated the applicability of the T2/FLAIR mismatch in an unselected cohort of patients with glioma that includes both lower-grade glioma and glioblastoma cases. We found that the T2/FLAIR-mismatch sign was confined to IDH-mutant (1p/19q non-codeleted) lower-grade gliomas and was absent in those cases of glioblastoma harboring the IDH mutation. These results suggest that the presence of T2/FLAIR-mismatch sign potentially allows to rule out IDH-wildtype tumors in the preoperative workup of gliomas. The previous study indicated that the addition of ADC and rCBV parameters can further improve the distinction of IDH-mutant 1p/19q non-codeleted from other molecular entities (IDH-mutant 1p/19q codeleted or IDH-wildtype tumors). Adding to this, our study now sheds light on the pathophysiology of the T2/FLAIR-mismatch sign, revealing significantly higher ADC and lower rCBV values for IDH-mutant gliomas with the presence of the T2/FLAIR-mismatch sign as compared to those IDH-mutant gliomas without. Although a previous study by Patel et al.^6^ did not find differences in overall survival between IDH-mutant glioma patients with versus without a T2/FLAIR-mismatch sign, both ADC and rCBV are well-known prognostic factors in patients with gliomas.^24–26^ Future studies should therefore investigate whether the quantifiable differences in the ADC and rCBV values in our study may also translate into a clinically measurable prognostic effect with a better outcome for those IDH-mutant gliomas with the presence of a T2/FLAIR-mismatch sign. In addition, our findings of lower ADC values in the FLAIR-hyperintense rim of cases with T2/FLAIR-mismatch sign are in line with reports from the pre-molecular era suggesting that the T2/FLAIR-mismatch sign may also implicate spatial differences in ADC values on DWI^27^ and potentially reflecting differences in cellularity and microenvironment within these tumors.

Although we did not observe a single false-positive case with a T2/FLAIR-mismatch sign in this large series of over 400 adult patients with newly diagnosed glioma—which is in line with the lower-grade glioma studies from Patel and Broen et al.^5,6^—a recent study reported contradicting findings.^28^ Specifically, Juratli et al.^28^ reported a relatively low specificity of only 76%, with however a relatively high sensitivity of 73% for identifying IDH-mutant (1p/19q non-codeleted) lower-grade gliomas. Whether these findings are due to a potentially less restrictive interpretation of the T2/FLAIR-mismatch sign in the study from Juratli et al. or due to intrinsic variations in the patient composition across the different studies remains uncertain. In our study, we defined the mismatch sign as a homogeneously/near-homogeneously hyperintense T2-w signal and simultaneously homogeneously/near-homogeneously hypointense signal on FLAIR with a complete/near-complete hyperintense peripheral rim on FLAIR.^5^ Strict adherence to this definition may explain the high specificity (100%) and low sensitivity (10.9%) of the T2/FLAIR-mismatch sign in our study. Future efforts should therefore not only focus on validating the T2/FLAIR-mismatch sign through a visual assessment on standard MRI, but also exploiting quantitative (radiomics) based approaches that potentially enable a more standardized assessment and may allow to overcome subjective interpretation by human readers. Nevertheless, given the evidence from the present and the majority of previous studies,^5,6,9^ the T2/FLAIR mismatch is the most appropriate imaging sign for identifying IDH-mutant (1p/19q non-codeleted) tumors.

Furthermore, we could show that there are differences in ADC values between patients who present the T2/FLAIR sign and patients who did not, which is consistent with recent publications describing the added value of adding advanced imaging parameters to distinguish between the different molecular subgroups.^26^ Our study has several limitations. First, this study followed a retrospective design and the assessment of the T2/FLAIR-mismatch sign was performed by 2 readers with variable experience, rather than exploiting quantitative (eg, radiomics) based approaches that could enable a more reproducible assessment. Secondly, the MRI data within the present study were derived from a single institution with all patients scanned at 3 T, which may render a standardized interpretation of the T2/FLAIR-mismatch sign easier and more reproducible in comparison to more heterogeneous MRI data (eg, derived from various institutions or field strengths). Even though we have a large patient population, the proportion of glioblastoma patients harboring the IDH mutation of all glioblastoma patients is only 1.7%, which is less than described in the literature, which is about 5%.^27^ This may be a possible limitation that none of the glioblastoma patients harboring the IDH mutation showed the T2/FLAIR-mismatch sign. Confirmatory studies with larger patient series are needed to confirm our results on lower ADC values in the rim of tumors with the presence of a T2/FLAIR-mismatch sign.

In conclusion, our study confirms the high specificity of the T2/FLAIR-mismatch sign for noninvasive identification of IDH-mutant 1p/19q non-codeleted gliomas, although sensitivity is low and applicability is limited to lower-grade gliomas. Whether the higher ADC and lower rCBV values in IDH-mutant gliomas with a T2/FLAIR-mismatch sign (as compared to those without) translate into a measurable prognostic effect requires investigation in future studies. Moreover, spatial differences in ADC values between the core and rim of tumors with a T2/FLAIR-mismatch sign potentially reflect specific distinctions in tumor cellularity and microenvironment.

## Supplementary Material

vdaa004_suppl_Supplementary_Table

## Notes

**Authorship Statement:** *Conceived and designed the research*: P.K., M.F., and K.N.N.T. *Acquired the data*: M.F., K.N.N.T., U.N., G.B., A.R., D.S., S.H., C.H.-M., A.U., J.D., A.v.D., W.W., M.B., and P.K. *Analyzed and interpreted the data*: M.F., K.N.N.T., P.K., W.W., and M.B. *Performed statistical analysis*: P.K. and M.F. *Handled funding and supervision*: P.K. *Drafted the manuscript*: P.K. and M.F. *Made critical revision of the manuscript for important intellectual content*: all authors.
