# Supplementary material for: T2/FLAIR-mismatch sign for noninvasive detection of IDH-mutant 1p/19q non-codeleted gliomas: validity and pathophysiology
Source: Neurooncol Adv. 2020 Jan 10;2(1):vdaa004. doi: 10.1093/noajnl/vdaa004 (PMC7212872; doi:10.1093/noajnl/vdaa004)
Supplement: vdaa004_suppl_Supplementary_Table [file vdaa004_suppl_supplementary_table.docx]

**Supplementary Table 1**. Characteristics of the final cohort distributed by integrated diagnosis including patient age at diagnosis and gender.

| **Tumor entity** | **n (%)** | **Age,**  median (IQR) | **Sex**  n (%) | |
| --- | --- | --- | --- | --- |
|  |  |  | Male | Female |
| Diffuse astrocytoma IDH-mut. (WHO °II) | 28 (6.9) | 43 (29-55) | 16 (57) | 12 (43) |
| Anaplastic astrocytoma IDH-mut. (WHO °III) | 38 (9.3) | 38 (30-47) | 24 (63) | 14 (37) |
| Anaplastic astrocytoma IDH-wild type (WHO °III) | 3 (0.7) | 50 (43-59) | 2 (67) | 1 (33) |
| Oligodendroglioma IDH-mut. 1p/19q codeleted (WHO °II) | 33 (8.1) | 51 (43-55) | 18 (55) | 15 (45) |
| Anaplastic oligodendroglioma IDH-mut. 1p/19q codeleted (WHO °III) | 11 (2.7) | 44 (37-53) | 6 (55) | 5 (45) |
| Glioblastoma IDH-wildtype (WHO °IV) | 282 (69.1) | 63 (56-72) | 156 (55) | 126 (45) |
| Glioblastoma IDH-mut. (WHO °IV) | 5 (1.2) | 39 (36-43) | 4 (80) | 1 (20) |
| Diffuse midline glioma H3-K27M-mut. (WHO °IV) | 8 (2.0) | 38 (20-55) | 2 (25) | 6 (75) |
| **Total** | **408** | **57** (47-69) | **228** (56) | **180** (44) |
